# 

**Published:** 1878-12-20

**Authors:** 


					﻿VOL. VIII.	DEC. 20, 1878.	No. 12.
£
W
03
W
w
r-H
o
Q
|*4
5S
p
o
k*
o
H
i-2
PS
tD
o
*“5
□Q
t—I
w
fa
O
CD
I—I
J
Q
W
H
H
□2
PS
Pu
cc
<3
W
cl
THE SOUTHERN
MEDICAL RECORD.
ft J^ONTHLY jJoUI\NAL OF PRACTICAL ^.edicine.
Terms : 82 OO per annum in advance. Club Rates, o copies
88.00. Single copies 20 cents.
“QUICQUID PR^CIPIES ESTO BREVIS.”
co
pc
o
w
H
8,
&
£
*
»—i
Q
>
H
i—i
O
CO
*
t?
U
o
h-H
o
r
i—i
H
W
£
co
co
O
H
H-<
Q
I—(
H
U
ESTABLISHED IN 1870
ATLANTA, GA.:
Sunny South Steam Publishing House.
Address R. C. WORD, M. D., Managing Editor, Atlanta, Ga.
				

